# The impact of occupational psychological hazards and metabolic syndrome on the 8-year risk of cardiovascular diseases—A longitudinal study

**DOI:** 10.1371/journal.pone.0202977

**Published:** 2018-08-27

**Authors:** Wei-Liang Chen, Chung-Ching Wang, Sheng-Ta Chiang, Ying-Chuan Wang, Yu-Shan Sun, Wei-Te Wu, Saou-Hsing Liou

**Affiliations:** 1 Division of occupational medicine, Division of fanily medicine, Department of Family and Community Medicine, Tri-Service General Hospital, National Defense Medical Center, Taipei, Taiwan, Republic of China; 2 National Institute of Environmental Health Science, National Health Research Institutes, Miaoli, Taiwan, Republic of China; 3 Department of Public Health, National Defense Medical Center, Taipei, Taiwan, Republic of China; Universidad Miguel Hernandez de Elche, SPAIN

## Abstract

There was little information concerning the combined effect of occupational psychosocial hazards such as long working hours, high job stress, and high fatigue on the risk of cardiovascular and cerebrovascular diseases (CVD). The aim of this study was to investigate the interaction among occupational psychosocial hazards and the impact of metabolic syndrome (MetS) on the risk of CVD among bus drivers. The Taiwan Bus Driver Cohort Study involving 1014 professional drivers was established in 2005 and comprehensively studied. The interactions among occupational psychosocial hazards and the impact of MetS on the risk of CVD were measured. A working pattern questionnaire, job stress questionnaires, the Swedish occupational fatigue inventory, the stress satisfaction offset score, biochemical measurements, and physical examinations were used to assess psychosocial hazards and the presence of metabolic syndrome. There were 707 eligible bus drivers with a mean age of 43.5years old. During the 8-years of follow-up, 77 drivers were diagnosed with CVD. Long working hours, high job stress, and high fatigue were associated with an increased risk of cardiovascular disease incidence in the multivariate analysis. There were synergistic effects among long working hours, high job stress, and high fatigue only in drivers with MetS. A combination of long working hours, high job stress, and high fatigue increased the risk of developing CVD in bus drivers with MetS.

## Introduction

The prevention of cardiovascular and cerebrovascular diseases (CVD) was still a major public health challenge worldwide despite modern medical advances. Psychosocial factors at work and health behaviors played a pivotal role in the pathogenesis and progression of CVD. Recently, research efforts had been increasingly concerned about the effect of occupational hazards on CVD. In a meta-analysis of 603,838 individuals, long working hours (≥55 hours per week) were associated with a 13% increase in coronary heart disease risk and a 33% increase in stroke risk [1]. In the Nurses’ Health Study, rotating night shift work was associated with a 4% increased risk of ischemic stroke for every 5 years[2]. Previously, 13 European epidemiologic cohort studies had reported that the association between job strain and coronary heart disease was significant for men and women, including those younger and older than 50 years of age, and at all levels of socioeconomic status[3]. Based on abovementioned evidence, overwork was a multifactorial, work-related psychosocial factor that interacted with environmental and lifestyle factors to cause CVD or karoshi. In the transportation industry, concurrent individual risk factors and work environmental risk factors among professional drivers contributed to cumulative CVD impact, such as age, metabolic syndrome, smoking, sedentary work, unconventional and irregular hours, shift work, long working hours, a tight delivery schedule, and job strain. Among approximately 120,000 Japanese workers from a national representative insurance organization, several business categories, including transportation, construction, and mining had the highest prevalence for metabolic syndrome (MetS) at 25.7%, 21.0%, and 20.5%, respectively[4]. Among them, metabolic Syndrome (MetS) is a cluster of metabolic abnormalities and is considered as a multi-morbid condition.

Metabolic syndrome (MetS), characterized by central obesity, glucose intolerance, atherogenic dyslipidemia, and fluctuations in blood pressure, was strongly associated with increased risk of coronary heart disease, cerebrovascular diseases, diabetes, and mortality[5, 6]. Patients with MetS were associated with a 2-fold increase in cardiovascular outcomes and a 1.5-fold increase in all-cause mortality compared with those without MetS[7]. Moreover, previous Norwegian HUNT Study mentioned that the association between metabolic syndrome and the risk of death from cardiovascular disease was stronger among persons with sedentary work and physically heavy work compared with persons with a lot of walking/lifting at work[8]. These findings implied that occupational physical activity or job categories may moderate the detrimental effects of MetS on CVD. Despite the tight link between MetS and CVD risk, little empirical evidence had been found that the psychosocial factors in workplace play which role in the development of MetS and CVD.

Importantly, light could be shed on psychosocial risks in workplace which was still largely uninvestigated. Therefore, studies on whether or not psychosocial factors contributes to or exacerbates CVD with/without MetS, could support a novel target for cardiovascular risk reduction in bus drivers. Thus, we performed a perspective cohort study to investigate the interaction of occupational psychosocial hazards with the risk of CVD among drivers with MetS.

## Methods

### Ethics statement

Our study protocol was approved by the institutional review board (IRB) of the National Health Research Institutes and Tri-Service General Hospital in accordance with the revised Helsinki Declaration. All subjects completed consent forms and agreed to participate in the survey after a detailed explanation of the protocol of the study.

### Study population

A total of 1,650 professional drivers from a largest transportation company were recruited in the Taiwan Bus Driver Cohort Study (TBDCS). After completion of informed consents, all participants received the basic and working related questionnaire and biochemistry tests. This cohort was linked to the Driving Hours Dataset during the period from 2005 to 2007[9]. At baseline, all 1,650 eligible drivers were followed up at the time of the evaluation and received comprehensive examinations. Our exclusion criteria included previous CVD history (n = 85), driving days < 100 days (n = 613), missing information on demographics (n = 23), and biochemical examinations (n = 222). To ensure some homogeneity of social background, all subjects were selected from the same bus company. Finally, 707 eligible drivers were followed up from 2005 to 2012.

### Definition of metabolic syndrome

The diagnosis of metabolic syndrome (MetS) was defined as the presence of three or more of the following components according to the Taiwan Health Promotion Administration of the Ministry of Health and Welfare in 2007[10]. Among the MetS criteria, the first component was waist circumference, which was replaced by BMI >27 kg/m^2^ in our study. Based on the receiver operating characteristic curve analysis from the Taiwan National Nutrition and Health Survey, a BMI cutoff of 27 kg/m^2^ was used to predict abdominal visceral obesity and waist circumference of 90 cm for men and 80 cm for women[11]^,^ [12]. The second component of MetS was blood pressure, which was determined to be abnormal by a systolic BP≥130 mm Hg, diastolic BP≥85 mm Hg, or self-reported hypertension. The third component was serum triglycerides (TGs), which was classified abnormal as serum TGs≥150 mg/dL (1.7 mmol/L). The fourth component was serum fasting glucose based on serum fasting glucose≥ 100 mg/dL or self-reported diabetes mellitus. The last component represented decreased serum high-density lipoprotein cholesterol (HDL) according to HDL <40 mg/dL (1.03 mmol/L) for men and <50 mg/dL (1.3 mmol/L) for women.

### Job stress assessment

We used the Chinese version of the Job Content Questionnaire (JCQ) to assess the psychosocial job characteristics of all participants[13]. The scores of the validated JCQ were based on the Demand-Control-Support model established by Karasek and Theorell[14]. Among the 22 items, the JCQ had a minimum set of questions for the assessment of the four factors, including job control, psychological demands, supervisor support, and co-worker support. A psychological demand score greater than 30 was classified as high job demand, and a decision latitude score that was greater 60 was categorized as high job control. The definition of high strain included high job demand and low job control, whereas the category of low strain was composed of low job strain (low job demand and high job control), an active job (high job demand and high job control), and a passive job (low job demand and low job control).

### Workplace stress evaluation tools

The questionnaires of Stress Satisfaction Offset Score (SSOS), a four-item scale created by Shain, is commonly used to evaluate the influence of workplace stressors in the workers. The four domains of SSOS include Demand Control model and the Effort Reward Imbalance model of workplace stress[15, 16]. There are control, effort, reward, and demand in the questionnaires. In terms of scoring scale in the SSOS questionnaires, total score ranges from -2 through to +2. A negative SSOS indicates that stress outweighs satisfaction, whereas a positive SSOS indicates that satisfaction outweighs stress[17].

### Dimensional assessment of fatigue questionnaire

The Swedish Occupational Fatigue Inventory (SOFI) is commonly used to assess the five domains of work-related perceived fatigue, including lack of energy, physical exertion, physical discomfort, lack of motivation, and sleepiness[18]. The participants scores their perceived intensity of fatigue based on 25 items.

### Sociodemographic variables

All eligible subjects were interviewed using a structured questionnaire, including questions regarding demographics (age, marital status, and education status), work conditions (work hours, years of driving experience, frequency of driving more than 12 hours a day, and schedule of rotating shifts), lifestyle habits (smoking, betel nut chewing, and drinking), and past medical histories. Long working hours were analyzed as a categorical variable (driving hours more than 12 hour/day and less than or equal to 12 hour/day). Body weight and height were evaluated using a digital scale, and BMI level was calculated as the subject's weight in kilograms divided by the square of their height in meters (kg/m^2^). We used a digital automatic sphygmomanometer to measure systolic and diastolic blood pressure in a sitting position after 10 to 15 minutes of rest. The blood testing was collected after 8 to 10 hours of overnight fasting to determine serum levels of high-density lipoprotein cholesterol, triglyceride and fasting blood glucose.

### Outcome measures

Each eligible subject was traced from the date of the baseline evaluation to the development of CVD or the end of the follow-up. The primary endpoint in our study was incident CVD, including ischemic heart disease, congestive heart failure, dissecting aneurysm of the aorta, serious cardiac arrhythmia, and cerebrovascular disease (ICD9: 390–459 and ICD10: I00-I99) based on the recognition criteria for overwork-related CVD in Taiwan [19, 20].

### Statistical analysis

All statistical analyses were examined using SPSS (Version 18.0 for Windows, SPSS, Inc., Chicago, IL, USA). Two-sided P-values < 0.05 were used to indicate significant differences. Concerning the association analysis, Cox proportional hazards regression was used to investigate the hazard ratios (HRs) for cardiovascular and cerebrovascular disease incidence. The potential confounders adjusted in the HRs analysis included age, BMI, education, drinking, smoking, and exercise. Five models were used to clarify the correlations among metabolic syndrome, work-related factors, and CVD incidences. Dichotomized occupational psychosocial hazards in theses analyses were long working hour (driving hour>12 hour/day vs ≤12 hour/day), high job stress (SSOS score < +2 vs +2), and high fatigue (SOFI ≥3.5 vs < 3.5). The five models analyzed include: Model 1: a single occupational psychosocial hazard was investigated for CVD incidences; Model 2: long working hours and high job stress were investigated for CVD incidence; Model 3: long working hours and high fatigue were investigated for CVD incidence; Model 4: high job stress and high fatigue were investigated for CVD incidences. Model 5: three variables, including long working hours, high job stress, and high fatigue, were investigated for CVD incidence. Finally, sensitivity analyses were performed to determine the robustness of the findings using different exclusion criteria. In the sensitivity analyses (S1 and S2 Tables) of 778 drivers, the exclusion definition of previous CVD history did not include hypertension (n = 71).

## Results

### Baseline characteristics

Demographic characteristics and biochemical indices were presented in Table 1. At study baseline, there were 327 drivers with MetS and 380 drivers without MetS (Non-MetS). The mean age of participants with MetS and Non-MetS was 43.8 and 43.2 years old, respectively. With comparison to participants with non-MetS, participants with MetS were older and had higher mean BMI, systolic blood pressure, diastolic blood pressure, total cholesterol, triglyceride, and fasting blood glucose.

**Table 1:** Demographic characteristics and biochemical indices among bus drivers and stratified by metabolic syndrome (n = 707).

Variables	All drivers (N = 707)	MetS (N = 327)	Non-MetS (N = 380)	*p-value*
Continuous variables				
Age (years), mean (SD)	43.5 (6.9)	43.8 (6.5)	43.2 (7.2)	0.285
BMI (kg/m^2^), mean (SD)	26.1 (4.0)	28.4 (3.8)	24.1 (3.0)	<0.001
Systolic blood pressure (mmHg), mean (SD)	126.9 (13.1)	132.2 (12.2)	122.4 (12.1)	<0.001
Diastolic blood pressure (mmHg), mean (SD)	80.9 (10.3)	85.1 (10.0)	77.3 (9.1)	<0.001
Total cholesterol (mg/dl), mean (SD)	203.5 (38.0)	210.1 (38.0)	197.8 (37.1)	<0.001
Triglyceride (mg/dl), mean (SD)	209.9 (181.3)	287.1 (226.0)	143.6 (87.9)	<0.001
HDL cholesterol (mg/dl), mean (SD)	37.7 (9.3)	33.6 (7.0)	41.2 (9.7)	<0.001
Fasting blood glucose (mg/dl), mean (SD)	102.7 (41.5)	115.2 (53.2)	91.9 (22.7)	<0.001
Categorical variables				
CVD after follow-up				0.012
Yes	77(10.9)	46(14.1)	31(8.2)	
No	630(90.1)	281(85.9)	349(91.8)	
Marital status, n (%)				0.899
Unmarried	123 (17.4)	59 (18.0)	64 (16.8)	
Married	506 (71.2)	233 (71.3)	273 (71.8)	
Others	78 (11.4)	35 (10.7)	43 (11.4)	
Education, n (%)				0.563
≤ Junior high school	206 (29.1)	93 (28.4)	113 (29.7)	
Senior high and vocational school	451 (63.8)	214 (65.4)	237 (62.4)	
University and College	50 (7.1)	20 (6.2)	30 (7.9)	
Cigarette smoking, n (%)				0.033
Never smokers	241 (34.1)	99 (30.3)	142 (37.4)	
Ex-smokers	47 (6.6)	17 (5.2)	30 (7.9)	
Current smokers	414 (59.3)	207 (64.5)	207 (54.7)	
Missing n = 5				
Alcohol use, n (%)				0.489
No	557 (82.4)	253 (77.4)	304 (80.0)	
Yes	146 (17.6)	71 (22.6)	75 (20.0)	
Missing n = 4				
Betel nut chewing				0.110
No	583 (82.5)	262 (80.1)	321 (84.5)	
Yes	123 (17.5)	65 (19.9)	58 (15.5)	
Missing n = 1				
Moderate exercise, n (%)				0.159
No	516 (72.9)	248 (75.8)	268 (70.5)	
Yes	181 (27.1)	76 (24.2)	105 (29.5)	
Missing n = 10				
Working hour, n (%)				0.134
≤ 12 hr	424 (59.9)	186 (56.9)	238 (62.6)	
> 12 hr	278 (40.1)	138 (43.1)	140 (37.4)	
Missing n = 5				
SSOS, n (%)				0.966
+ 2 ~ +1	161 (22.8)	66 (20.2)	95 (25.0)	
-2 ~ 0	544 (77.2)	261 (79.8)	283 (75.0)	
Missing n = 2				
SOFI, n (%)				0.610
< 3.0	316 (44.7)	143 (43.7)	173 (45.5)	
≥3.0	390 (55.3)	184 (56.3)	206 (54.2)	
Missing n = 1				
Shift work, n (%)				0.083
No	127 (17.9)	50 (15.3)	77 (20.3)	
Yes	579 (82.1)	277 (84.7)	302 (79.5)	
Missing n = 1				

SD, standard deviation; MetS, metabolic syndrome; BMI, body mass index; HDL, high-density lipoprotein; SSOS, Stress Satisfaction Offset Score; SOFI, Swedish Occupational Fatigue Inventory

### Metabolic syndrome, psychosocial hazards, and CVD

During the 8-years of follow-up, there were 77 drivers (10.9%) developed CVD (Table 1). The crude hazard ratio among the individuals with MetS was 1.77 (P value = 0.014) compared to those without MetS (Table 2). For long working hours, working hour >12 hour/ day was significantly associated with an increased risk of CVD incidence in the multivariate analysis (HR = 1.76, 95% CI = 1.11–2.79). In terms of high job stress, risk for CVD incidence was not significantly increased among the drivers with high job stress (multivariate analysis HR = 1.20, 95% CI = 0.67–2.14) when compared with those with low job stress (scores of SSOS +2~+1). We observed a borderline significant trend association between high fatigue (elevated scores of SOFI) and the risk of CVD.

**Table 2:** Unadjusted and adjusted hazard ratios of work patterns and psychosocial hazards on CVD in bus drivers (n = 707).

		Univariate Analysis				Multivariate Analysis ^a^^.^			
	*Independent variables* ^*b*^	*HR*	*95% CI*		*p-value*	*HR*	*95% CI*		*p-value*
1	Metabolic syndrome (Ref.< No)								
	Yes	1.77	1.12	2.79	0.014	1.38	0.80	2.36	0.247
2	Long working hours (Ref. Working hour ≤12 hour/ day)								
	Working hour >12 hour/ day	1.93	1.23	3.02	0.004	1.76	1.11	2.79	0.016
3	Shift work (Ref. No shift work)								
	Shift work	0.74	0.43	1.28	0.281	0.68	0.39	1.18	0.172
4	Job Content Questionnaire (Ref. Low strain)								
	High strain	1.17	0.71	1.94	0.538	1.08	0.65	1.80	0.772
5	Stress Satisfaction Offset Score (Ref. = +2~+1)								
	-2 ~ 0	1.38	0.78	2.42	0.270	1.20	0.67	2.14	0.539
6	Swedish Occupational Fatigue Inventory (Ref. <3)								
	> = 3	1.51	0.95	2.39	0.080	1.58	0.98	2.55	0.060

CVD, cardiovascular and cerebrovascular diseases; HR, hazard ratios; CI, confidence interval; BMI, body mass index.

^a.^ Adjusted for age, BMI, education, drinking, smoking, and exercise.

^b.^ Each independent variable (1–6) was solely included in the models

### Relationship among metabolic syndrome, occupational psychosocial hazards, and CVD

In the univariate analysis (model 1 of Table 3), only long working hours were significantly associated with increased risk of CVD among participants with MetS (multivariate analysis HR = 1.65, 95% CI = 1.03–2.66). The univariate and multivariate analyses were performed to determine the association between long working hours and high job stress (model 2 of Table 3); the adjusted HR ratio of participants with both long working hours and high job stress compared with those with neither long working hours nor high job stress was 2.17 (95% CI = 0.99–4.79, P value = 0.054). Compared to the group with neither long working hours nor high fatigue (model 3 of Table 3), the adujsted HR for CVD was 2.31 (95% CI = 1.26–4.23, P value = 0.007) among those with both long working hours and high fatigue. The adjusted HR ratio of participants with both high job stress and high fatigue compared to those with neither high job stress nor high fatigue was 2.07 (95% CI = 0.90–4.73, P value = 0.086). In the final model, the univariate and multivariate analysis HRs of MetS participants with three occupational psychosocial hazards (model 5 of Table 3) were 4.10 (95% CI = 1.42–11.08, P value = 0.009) and 3.71 (95% CI = 1.26–10.91, P value = 0.017), respectively, which were higher than the HRs of participants with two psychosocial hazards. Our results still remained robust to sensitivity analyses using the different exclusion definitions (S1 Table).

**Table 3:** Hazard ratios f of interactions among psychosocial hazards or on CVD classified by psychosocial hazards in participants (n = 707).

Dependent variable	Univariate Analysis				Multivariate Analysis			
Model 1	HR	95%CI		*p-value*	HR	95%CI		*p-value*
Long working hours	1.76	1.11	2.81	0.017	1.65	1.03	2.66	0.037
High job stress	1.03	0.56	1.87	0.936	0.94	0.51	1.74	0.852
High fatigue	1.31	0.81	2.12	0.280	1.43	0.87	2.36	0.160
MetS	1.69	1.07	2.67	0.025	1.33	0.77	2.29	0.308
Model 2								
Ref: Working hours < = 12 hrs and SSOS = +2								
Either long working hours or high job stress only	1.80	0.83	3.89	0.135	1.64	0.76	3.57	0.210
Both long working hours and high job stress	2.48	1.14	5.37	0.022	2.17	0.99	4.79	0.054
MetS	1.69	1.07	2.67	0.025	1.33	0.77	2.30	0.302
Model 3								
Ref: Working hours < = 12 hr & SOFI<3								
Either long working hours or high fatigue only	1.27	0.71	2.28	0.419	1.35	0.75	2.43	0.321
Both long working hours and high fatigue	2.29	1.28	4.09	0.005	2.31	1.26	4.23	0.007
MetS	1.69	1.07	2.67	0.025	1.32	0.77	2.26	0.321
Model 4								
Ref: SSOS = +2 & SOFI<3								
Either high job stress or high fatigue only	2.10	0.92	4.77	0.078	1.90	0.83	4.35	0.130
Both high job stress and high fatigue	2.15	0.96	4.83	0.064	2.07	0.90	4.73	0.086
MetS	1.70	1.08	2.69	0.022	1.32	0.76	2.27	0.321
Model 5								
Ref: Working hours < = 12 hr & SSOS = +2 & SOFI<3								
One of three variables (long working hours, high job stress, or high fatigue)	2.76	0.95	8.02	0.062	2.43	0.83	7.08	0.105
Two of three variables (long working hours, high job stress, or high fatigue)	2.66	0.92	7.67	0.070	2.57	0.89	7.45	0.082
All of three variables (long working hours, high job stress, or high fatigue)	4.10	1.42	11.80	0.009	3.71	1.26	10.91	0.017
MetS	1.69	1.07	2.67	0.025	1.31	0.76	2.26	0.331

Long working hours = driving hour>12 hour/ day; High job stress = SSOS_scores < +1; High fatigue = SOFI > = 3.

CVD, cardiovascular and cerebrovascular diseases; HR, hazard ratios; CI, confidence interval; MetS, metabolic syndrome; SSOS, Stress Satisfaction Offset Score; SOFI, Swedish Occupational Fatigue Inventory.

^a.^ Adjusted for age, BMI, education, drinking, smoking, and exercise.

In the Table 4, we showed the different combinations of individual psychosocial hazards and metabolic syndrome for predicting CVD. Compared with those without any psychosocial hazards, there were no significant associations with CVD among non-MetS participants with 1–3 occupational psychosocial hazards. Notably, multivariate analysis HRs of MetS participants with two (long working hours+ high fatigue) and three occupational psychosocial hazards were 10.91 (95% CI = 1.02–56.30, P value = 0.004) and 3.72 (95% CI = 1.02–13.56, P value = 0.046), respectively. In the sensitivity analyses (S2 Table), our results were still robust to using the different exclusion definitions.

**Table 4:** Combinations of individual psychosocial hazards and metabolic syndrome for predicting CVD (n = 707).

					Univariate Analysis				Multivariate Analysis ^a^^.^			
Combinations	LWH	HJS	HF	MetS	*HR*	*95%CI*		*p-value*	*HR*	*95%CI*		*p-value*
1	**-**	**-**	**-**	**-**	Reference				Reference			
2	**+**	**-**	**-**	**-**	2.95	0.49	17.66	0.237	2.38	0.39	14.66	0.349
3	**-**	**+**		**-**	1.38	0.33	5.78	0.662	1.22	0.29	5.20	0.788
4	**-**	**-**	**+**	**-**	1.74	0.29	10.49	0.544	1.99	0.33	12.15	0.458
5	**+**	**+**		**-**	0.61	0.06	5.83	0.664	0.66	0.07	6.42	0.721
6	**-**	**+**	**+**	**-**	2.27	0.60	8.63	0.227	2.31	0.60	8.91	0.223
7	**+**		**+**	**-**	6.75	0.70	65.09	0.099	6.94	0.69	69.77	0.100
8	**+**	**+**	**+**	**-**	2.58	0.69	9.62	0.158	2.46	0.64	9.43	0.190
9	**+**	**-**	**-**	**+**	2.53	0.26	24.37	0.422	2.19	0.23	21.38	0.499
10	**-**	**+**	**-**	**+**	3.45	0.95	12.60	0.061	2.62	0.69	9.95	0.158
11	**-**	**-**	**+**	**+**	3.56	0.59	21.33	0.165	2.97	0.48	18.28	0.241
12	**+**	**+**	**-**	**+**	4.46	1.15	17.29	0.031	3.24	0.80	13.14	0.100
13	**-**	**+**	**+**	**+**	1.48	0.35	6.20	0.595	1.30	0.30	5.60	0.723
14	**+**	**-**	**+**	**+**	12.01	2.39	60.43	0.003	10.91	2.11	56.30	0.004
15	**+**	**+**	**+**	**+**	4.76	1.39	16.34	0.013	3.72	1.02	13.56	0.046

LWH, long working hours; HJS, high job stress; HF, high fatigue; MetS, metabolic syndrome

^a.^ Adjusted for age, BMI, education, drinking, smoking, and exercise.

## Discussion

To the best of our knowledge, our study was the first investigation to examine the combined effect of metabolic syndrome and occupational psychosocial hazards on the risk of CVD. Thus, we sought to describe an approach to evaluate the status of overwork using by multiple clustered traits. In this 8-year longitudinal cohort study, we found that the risk of CVD was highest among the MetS participants who had long working hours, high job stress, and high fatigue. However, no evidence of associations between occupational psychosocial hazards and CVD was observed in the subjects without MetS. There was a linear increase in the HR among MetS participants with increased numbers of occupational psychosocial hazards. Among three occupational psychosocial hazards, long working hours were most associated with an elevated risk of CVD.

For detrimental effect of long working hours, our study finding was concordant with the findings of several previous studies. The results from several prospective observational studies suggested an approximately 40% increase in the risk of CHD among employees with long working hours[21]. In a systematic review of epidemiological evidence, long working hours had significant adverse effects on many health outcomes, including depressive state, anxiety, sleep condition, and coronary heart disease[22]. Moreover, employees who work long hours have an elevated risk of stroke than those who work standard hours[1]. There was one plausible explanation for this. Sleep deprivation and fragmentation caused by long working hours increased the risk of type 2 diabetes through β-cell dysfunction and insulin resistance, indirectly leading to accelerate insult to vascular endothelial cells and an increased risk of CVD[23].

High job stress was linked with a broader spectrum of diseases via an unhealthy lifestyle and obesity, including type 2 diabetes, coronary heart disease, and stroke[24, 25]. Meta-analysis of individual participant data from over 47,000 participants showed that the association between job strain and elevated cardiovascular risks was attributable to the higher prevalence of diabetes, smoking, and physical inactivity among those reporting job strains[26]. In the cross-sectional data of 46,573 Finnish public sector employees, an association between higher work stress and lower leisure time physical activity was observed[27]. High stress attenuated the willingness or the ability of employees to engage in regular exercise and other physical activity[28]. Another possible pathophysiological mechanism underlying the association between job strain and CVD was attributed to an imbalance of the autonomic nervous system and elevated catecholamines. Emerging evidence had shown that high strain jobs were reported to be significantly associated with decreased vagal tone indicators[29, 30]. It was suggested that reduced activity of the parasympathetic nervous system increased the risk of coronary heart disease[31].

In terms of high fatigue, MetS participants with high fatigue had a higher risk of CVD compared with those without three occupational psychosocial hazards. Consistent with our findings, Appels et al observed that undue fatigue was a frequent premonitory symptom of cardiac events. They delineated a triad of symptoms as vital exhaustion (VE) or exhaustion, including excessive fatigue, feelings of demoralization, and increased irritability[32]. In a meta-analysis of 17 studies concerning VE/exhaustion, a significant association between VE/exhaustion and CVD events remained after adjusting for a number of clinical and psychosocial factors[33]. The psychosocial consequences of fatigue were becoming increasingly addressed because high levels of fatigue, as measured by the Short Form 36 vitality domain, were associated with increased mortality in the general population[34]. Collectively, it was possible that fatigue or VE/exhaustion was a potential underlying psychophysiological mediator of CVD.

Even through this body of research had the undeniable merit of offering valuable insights into the interaction between MetS and psychosocial hazards, it also some limitations. The first limitation concerned the assessments used in the current study. Although there were many work-related psychosocial hazards that had a substantial impact on CVD outcomes, we tried to adopted several valuable questionnaires to evaluate these hazards. Second, the generalizability of the results to other populations with different occupations may be limited since all participants in the present study were drivers. Third, the interaction between MetS and changes in psychosocial hazards over time was not analyzed, because the pertinent variables were measured only once at enrollment into the study. Last, all measures were only taken once at baseline but not repeated measures during follow-up period. Moreover, the interpretation of our observations was limited because of absence of long-term changes in psychosocial hazards and cardiometabolic risks.

## Conclusion

Our findings clearly supported the notion that a combination of long working hours, high job stress, and high fatigue in MetS participants increased the risk of developing CVD. These results underscored the importance of recognizing these occupational psychosocial hazards in the drivers with MetS, especially combined with long working hours, high job stress, and high fatigue. It was tempting to speculate that since metabolic syndrome insulted cardiovascular healthy and ensuing psychosocial hazards could contribute to the deterioration of vascular function in individuals predisposed for CVD. Psychosocial interventions in the workplace may have beneficial effects on CVD morbidity and mortality. Further research might extend the investigation of psychosocial hazards to different occupations to examine the impact of MetS on CVD or mortality.

## Supporting information

S1 Table Hazard ratios f of interactions among psychosocial hazards or on CVD classified by psychosocial hazards in participants. (DOCX)

S2 Table Combinations of individual psychosocial hazards and metabolic syndrome for predicting CVD. (DOCX)
