# Supplementary material for: The impact of occupational psychological hazards and metabolic syndrome on the 8-year risk of cardiovascular diseases—A longitudinal study
Source: PLoS One. 2018 Aug 27;13(8):e0202977. doi: 10.1371/journal.pone.0202977 (PMC6110510; doi:10.1371/journal.pone.0202977)
Supplement: S1 Table — (DOCX) [file pone.0202977.s001.docx]

Supplement Table 1. Hazard ratios of psychosocial hazards or on CVD classified by psychosocial hazards in participants (n=778)

| **Dependent variable** | **Univariate Analysis** | | | | **Multivariate Analysis** | | | |
| --- | --- | --- | --- | --- | --- | --- | --- | --- |
| Model 1 | HR | 95%CI | | *p-value* | HR | 95%CI | | *p-value* |
| Long working hours | 1.75 | 1.25 | 2.45 | 0.001 | 1.77 | 1.16 | 2.70 | 0.008 |
| High job stress | 0.93 | 0.61 | 1.42 | 0.210 | 0.93 | 0.61 | 1.43 | 0.744 |
| High fatigue | 1.21 | 0.86 | 1.72 | 0.279 | 1.42 | 0.99 | 2.03 | 0.058 |
| MetS | 2.41 | 1.69 | 3.42 | <0.001 | 1.77 | 1.16 | 2.70 | 0.008 |
| Model 2 |  |  |  |  |  |  |  |  |
| Ref: Working hours <= 12 hrs and SSOS =+2 | | | | | | | | |
| Either long working hours or high job stress only | 1.06 | 0.65 | 1.74 | 0.818 | 1.08 | 0.66 | 1.78 | 0.762 |
| Both long working hours and high job stress | 1.77 | 1.09 | 2.86 | 0.021 | 1.75 | 1.07 | 2.86 | 0.026 |
| MetS | 2.42 | 1.70 | 3.44 | <0.001 | 1.82 | 1.19 | 2.77 | 0.006 |
| Model 3 |  |  |  |  |  |  |  |  |
| Ref: Working hours <= 12 hr & SOFI<3 | | | | | | | | |
| Either long working hours or high fatigue only | 1.46 | 0.96 | 2.22 | 0.077 | 1.56 | 1.02 | 2.39 | 0.039 |
| Both long working hours and high fatigue | 2.10 | 1.37 | 3.23 | 0.001 | 2.31 | 1.48 | 3.60 | 0.000 |
| MetS | 2.40 | 1.69 | 3.41 | <0.001 | 1.74 | 1.15 | 2.66 | 0.010 |
| Model 4 |  |  |  |  |  |  |  |  |
| Ref: SSOS =+2 & SOFI<3 |  |  |  |  |  |  |  |  |
| Either high job stress or high fatigue only | 1.71 | 0.98 | 2.97 | 0.058 | 1.73 | 0.99 | 3.03 | 0.053 |
| Both high job stress and high fatigue | 1.69 | 0.98 | 2.92 | 0.059 | 1.91 | 1.09 | 3.34 | 0.024 |
| MetS | 2.43 | 1.71 | 3.45 | <0.001 | 1.77 | 1.16 | 2.69 | 0.008 |
| Model 5 |  |  |  |  |  |  |  |  |
| Ref: Working hours <= 12 hr & SSOS =+2 & SOFI<3 | | | | | | | | |
| One of three variables (long working hours, high job stress, or high fatigue) | 1.84 | 0.94 | 3.59 | 0.074 | 1.93 | 0.98 | 3.78 | 0.056 |
| Two of three variables (long working hours, high job stress, or high fatigue) | 1.82 | 0.94 | 3.51 | 0.075 | 1.96 | 1.01 | 3.81 | 0.047 |
| All of three variables (long working hours, high job stress, or high fatigue) | 2.69 | 1.39 | 5.19 | 0.003 | 3.03 | 1.54 | 5.94 | 0.001 |
| MetS | 2.40 | 1.69 | 3.41 | <0.001 | 1.76 | 1.15 | 2.68 | 0.009 |

Long working hours= driving hour>12 hour/ day; High job stress= SSOS_scores < +1; High fatigue= SOFI >=3.

CVD, cardiovascular and cerebrovascular diseases; HR, hazard ratios; CI, confidence interval; MetS, metabolic syndrome; SSOS, Stress Satisfaction Offset Score; SOFI, Swedish Occupational Fatigue Inventory.

^a.^ Adjusted for age, BMI, education, drinking, smoking, and exercise.
