# Supplementary material for: The impact of occupational psychological hazards and metabolic syndrome on the 8-year risk of cardiovascular diseases—A longitudinal study
Source: PLoS One. 2018 Aug 27;13(8):e0202977. doi: 10.1371/journal.pone.0202977 (PMC6110510; doi:10.1371/journal.pone.0202977)
Supplement: S2 Table — (DOCX) [file pone.0202977.s002.docx]

Supplement Table 2 combinations of individual psychosocial hazards and metabolic syndrome for predicting CVD (n=778)

|  |  |  |  |  | Univariate Analysis | | | |  | Multivariate Analysis ^a.^ | | | |
| --- | --- | --- | --- | --- | --- | --- | --- | --- | --- | --- | --- | --- | --- |
| Combinations | LWH | HJS | HF | MetS | HR | 95%CI | | *p-value* |  | HR | 95%CI | | *p-value* |
| 1 | **-** | **-** | **-** | **-** | Reference |  |  |  |  | Reference |  |  |  |
| 2 | **+** | **-** | **-** | **-** | 2.26 | 0.41 | 12.34 | 0.347 |  | 2.23 | 0.40 | 12.40 | 0.359 |
| 3 | **-** | **+** |  | **-** | 1.04 | 0.28 | 3.89 | 0.951 |  | 1.01 | 0.27 | 3.82 | 0.983 |
| 4 | **-** | **-** | **+** | **-** | 1.93 | 0.43 | 8.65 | 0.392 |  | 2.73 | 0.60 | 12.36 | 0.193 |
| 5 | **+** | **+** |  | **-** | 1.76 | 0.44 | 7.06 | 0.424 |  | 2.13 | 0.53 | 8.60 | 0.287 |
| 6 | **-** | **+** | **+** | **-** | 2.29 | 0.73 | 7.23 | 0.157 |  | 2.74 | 0.86 | 8.73 | 0.089 |
| 7 | **+** |  | **+** | **-** | 5.11 | 0.57 | 45.82 | 0.145 |  | 4.46 | 0.49 | 40.72 | 0.185 |
| 8 | **+** | **+** | **+** | **-** | 3.11 | 1.03 | 9.42 | 0.045 |  | 3.65 | 1.19 | 11.27 | 0.024 |
| 9 | **+** | **-** | **-** | **+** | 5.49 | 1.23 | 24.58 | 0.026 |  | 4.30 | 0.95 | 19.60 | 0.059 |
| 10 | **-** | **+** | **-** | **+** | 4.44 | 1.51 | 13.09 | 0.007 |  | 3.76 | 1.24 | 11.38 | 0.019 |
| 11 | **-** | **-** | **+** | **+** | 8.75 | 2.63 | 29.14 | 0 |  | 7.94 | 2.33 | 27.11 | 0.001 |
| 12 | **+** | **+** | **-** | **+** | 6.91 | 2.3 | 20.73 | 0.001 |  | 4.80 | 1.55 | 14.88 | 0.007 |
| 13 | **-** | **+** | **+** | **+** | 2.53 | 0.81 | 7.87 | 0.108 |  | 2.34 | 0.74 | 7.40 | 0.148 |
| 14 | **+** | **-** | **+** | **+** | 8.32 | 1.85 | 37.51 | 0.006 |  | 7.18 | 1.57 | 32.94 | 0.011 |
| 15 | **+** | **+** | **+** | **+** | 6.36 | 2.25 | 17.99 | 0.001 |  | 5.62 | 1.92 | 16.48 | 0.002 |

LWH, long working hours; HJS, high job stress; HF, high fatigue; MetS, metabolic syndrome

^a.^ Adjusted for age, BMI, education, drinking, smoking, and exercise.
